# Sustained-Release and pH-Adjusted Alginate Microspheres-Encapsulated Doxorubicin Inhibit the Viabilities in Hepatocellular Carcinoma-Derived Cells

**DOI:** 10.3390/pharmaceutics13091417

**Published:** 2021-09-07

**Authors:** Cheng-Tang Pan, Ruei-Siang Yu, Chih-Jung Yang, Lih-Ren Chen, Zhi-Hong Wen, Nai-Yu Chen, Hsin-You Ou, Chun-Yen Yu, Yow-Ling Shiue

**Affiliations:** 1 Institute of Precision Medicine, National Sun Yat-Sen University, Kaohsiung 80424, Taiwan; pan@mem.nsysu.edu.tw (C.-T.P.); haylie65604@gmail.com (N.-Y.C.); 2 Department of Mechanical and Electro-Mechanical Engineering, National Sun Yat-Sen University, Kaohsiung 80424, Taiwan; karenyang@mem.nsysu.edu.tw; 3 Department of Pharmacy, Kaohsiung Armed Forces General Hospital, Kaohsiung 80424, Taiwan; shung804@gmail.com; 4 Division of Physiology, Livestock Research Institute, Council of Agriculture, Tainan 71246, Taiwan; lrchen@mail.tlri.gov.tw; 5 Department of Biotechnology and Bioindustry Sciences, National Cheng Kung University, Tainan 701401, Taiwan; 6 Department of Marine Biotechnology and Resources, National Sun Yat-Sen University, Kaohsiung 80424, Taiwan; wzh@mail.nsysu.edu.tw; 7 Liver Transplantation Program and Departments of Diagnostic Radiology and Surgery, Kaohsiung Chang Gung Memorial Hospital, and Chang Gung University College of Medicine, Kaohsiung 83301, Taiwan; ouhsinyou@gmail.com; 8 Institute of Biomedical Sciences, National Sun Yat-Sen University, Kaohsiung 80424, Taiwan

**Keywords:** sodium alginate, microsphere, ultrasonic atomization, Dox, pH-adjusted

## Abstract

The objective of this study aimed to develop biodegradable calcium alginate microspheres carrying doxorubicin (Dox) at the micrometer-scale for sustained release and the capacity of pH regulatory for transarterial chemoembolization. Ultrasonic atomization and CaCl_2_ cross-linking technologies were used to prepare the microspheres. A 4-by-5 experiment was first designed to identify imperative parameters. The concentration of CaCl_2_ and the flow rate of the pump were found to be critical to generate microspheres with a constant volume median diameter (~39 μm) across five groups with different alginate: NaHCO_3_ ratios using each corresponding flow rate. In each group, the encapsulation efficiency was positively correlated to the Dox-loading %. Fourier-transform infrared spectroscopy showed that NaHCO_3_ and Dox were step-by-step incorporated into the calcium alginate microspheres successfully. Microspheres containing alginate: NaHCO_3_ = 1 exhibited rough and porous surfaces, high Young’s modulus, and hardness. In each group with the same alginate: NaHCO_3_ ratio, the swelling rates of microspheres were higher in PBS containing 10% FBS compared to those in PBS alone. Microspheres with relatively high NaHCO_3_ concentrations in PBS containing 10% FBS maintained better physiological pH and higher accumulated Dox release ratios. In two distinct hepatocellular carcinoma-derived cell lines, treatments with microspheres carrying Dox demonstrated that the cell viabilities decreased in groups with relatively high NaHCO_3_ ratios in time- and dose-dependent manners. Our results suggested that biodegradable alginate microspheres containing relatively high NaHCO_3_ concentrations improved the cytotoxicity effects in vitro.

## 1. Introduction

Hepatocellular carcinoma (HCC) is one of the foremost causes of tumor-associated mortality worldwide and the incidence continues to increase [1]. Regrettably, HCC is usually diagnosed at intermediate or advanced stages while merely palliative remedies could be used, leading to poor overall survival. Transarterial chemoembolization (TACE) is a frequently recommended treatment for asymptomatic, multifocal, and/or large HCC devoid of macrovascular invasion or metastasis [2,3]. TACE intends to induce tumor neurosis, resulting in cytotoxic effects along with ischemia in the tumor tissue. Chemoembolization, especially using Doxorubicin (Dox), improved the survival of stringently selected patients with unresectable hepatocellular carcinoma [2]. 

Numerous types of microspheres including temporary or permanent are commercially available for TACE. The most commonly used are DC Bead™ [4] and HapaSphere™ [3] with the major component of polyvinyl alcohol (PVA), which were approved by the Food and Drug Administration (FDA, Hampton, VA, USA) as are biocompatible yet non-biodegradable [5]. The type and the size of the Dox-loaded microspheres decide drug release effects in vitro [6]. For local recurrence after TACE [7], re-TACE is a therapeutic option. Moreover, for patients who are suitable for liver transplantation, preventing artery occlusion is necessary [8]. Thus, the fabrication of biodegradable microspheres to prevent permanent embolization, compress arterial walls and normal organs by drug-loaded agents, as well as postoperative complications, are extremely important. 

Several biodegradable and biocompatible polymers including gelatin [9], chitosan [10], chitooligosaccharide [11], and sodium alginate [12,13,14] have been reported to formulate microspheres for TACE. As a low-priced and non-toxic polyanionic polysaccharide, alginate displays characteristics including highly biocompatible and hydrophilic, ease of gelation, inert nature, ease of availability, and a feasible method of synthesis. Therefore, it is an excellent choice for researchers to develop platforms for tissue engineering and drug delivery [15,16]. It is also frequently used as a viscosifier, stabilizer, or gelling agent in food, textile, pharmaceutical, and biotechnological industries [17]. Additional details are documented in ‘modified alginate copolymer, alginate nanoparticle, and applications thereof’ [US20190367656A1, Kumar J. (2018), https://patents.google.com/patent/US20190367656A1/en, 2 September 2021]. Moreover, Dox-loaded alginate microspheres showed a delayed release of the drug in the liver, extending the function time and maintaining the Dox concentration after embolization [18].

An increase in the rate of glucose uptake and preferential production of lactate, even in the presence of oxygen, also known as the ‘Warburg Effect’ [19,20], is prevalent in human cancer [21]. Accordingly, the increase of intratumoral lactate and its secretion in the tumor microenvironment resulting in lactate acidosis becomes an essential element in cancer progression and treatment [22,23], including hepatocellular carcinoma [24]. Compared to the conventional methods to prepare microspheres, the water-based ion cross-linking technique bestows distinguishing advantages. In order to fabricate alginate microspheres-encapsulated Dox with the spherical integrity for sustained-release, we previously used a dripping-cross-linking method to produce ~2 mm microspheres [13]. In this study, we aimed to promote the process to improve sustained release Dox microspheres at a micrometer-scale, as well as to incorporate a pH-adjusting agent, NaHCO_3_, for TACE.

## 2. Materials and Methods

### 2.1. Chemicals and Reagents

Sodium alginate (C_6_H_7_NaO_6_)_n_, calcium chloride (CaCl_2_) (Sigma-Aldrich, St. Louis, MO, USA), sodium bicarbonate (NaHCO_3_) (J. T. Baker, Phillipsburg, NJ, USA), ethyl acetate (CH_3_COOCH_2_CH_3_) (Merck, Darmstadt, Germany), and Dox (Concord Biotech Limited, Gujarat, India) were obtained. For cell culture, Dulbecco’s Modified Eagle Medium (DMEM, for Huh-7 cells), Minimum Essential Medium (MEM, for Hep-3B cells), and antibiotics (10,000 IU/mL penicillin and 10,000 IU/mL streptomycin) were purchased from HyClone™ Laboratories Inc. (Logan, UT, USA). Fetal Bovine Serum (FBS) and trypsin-EDTA were acquired from Gibco/Thermo Fisher Scientific (Carlsbad, CA, USA). All solutions were prepared with autoclaved Mini-Q ultrapure water. Solutions of sodium alginate, CaCl_2_ (for cross-linking), ethyl acetate (the oil-phase solution), and Dox were prepared as 2.2 wt%, 7wt%, 10 wt%, and 2 mg/mL, respectively. 

### 2.2. Preparation of the Dox Calcium Alginate Microspheres Containing NaHCO_3_

The instruments including the infusion pump and height gage were used. A dripping-cross-linking method was applied to fabricate the calcium alginate microspheres. Supplementary Figure S1 displays the experimental setups where the mixture (sodium alginate, NaHCO_3_ and, Dox) was located in a syringe and the syringe was squeezed by an infusion pump (KDS100, kdScientific, Holliston, MA, USA). The ratios of sodium alginate to NaHCO_3_ were designed as 8:1, 4:1, 2:1, 1:1, and 1:2. All solutions were prepared with sterilized Mini-Q ultrapure water. An ultrasonic atomization (8700-48H, Sono-Tek, Milton, NY, USA) with 43 kHz high-frequency vibrations (12 V and 0.56 A power output) was used to atomize the admixture into micron-sized droplets, dropped into the calcium chloride (CaCl_2_) containing 10 wt% ethyl acetate (oil phase, to avoid aggregation) for crosslinking and continuously stirred for 3 h for solidification. Afterwards, the microspheres were collected by using a 10-micron/1500 mesh nylon filter (Shijiazhuang, Hebei, China), washed with sterilized Mini-Q ultrapure water to remove extra ethyl acetate, sanitized with 75% ethanol for 10 min, rewashed with sterilized water, and stored in CaCl_2_ (0.6 wt%) solution at room temperature. All procedures were performed in a laminar flow hood.

### 2.3. Experimental Design and Analysis

Our previous study (Pan et al. 2020) identified 4 critical parameters impacting the particle size of microspheres. Therefore, a 4-by-5 (20 in total) experiment was initially designed to identify the significance of each parameter to the particle size of the microspheres (Supplementary Table S1). The code and level of all parameters designated as Supplementary Table S2, experiment 1 was selected as the reference group. Thus, a total of 17 experiments were performed to identify the significance of each parameter (the concentrations of sodium alginate and CaCl_2_ (wt%), the stirring speed (rpm), and the flow rate of the pump (mL/h)). For each experiment, one parameter was changed while others were fixed (Supplementary Table S3), and results were subjected to ANOVA with post hoc tests to identify critical parameters. For each experiment, ≥3 independent trials were carried out to produce microspheres. An optical microscope (LV-UEPI, Nikon, Minato-ku, Tokyo, Japan) with MultiCam EZ M9 software (version 2.2, BAITE, Kwun Tong, Kln., Hong Kong) was used to measure the particle diameters of 3 subsets of the microspheres, which were randomly sampled from each trail. The volume median diameter (Dv(50)) was determined next. 

### 2.4. Drug Encapsulation and Loading %

Both Dox and sodium alginate are water-soluble, hence Dox can be dissolved in sodium alginate solution. The mixture of Dox-sodium alginate was then ionically crosslinked with calcium ions in CaCl_2_ solution to form a three-dimension network of hydrogel spheres, thus Dox was encapsulated. Exactly 10 mL of the Dox-loaded sodium alginate solution (2 mg/mL) was placed in a syringe to prepare the Dox-loaded microspheres. After the calcium alginate microspheres were fabricated, the Dox remains of the CaCl_2_ solution and the Dox-loaded sodium alginate solution left in the syringe were collected, measured, and converted to weight. Drug residues in the syringe after dipping and a gelation bath were both considered (see also Section 2.5.2). Therefore, encapsulation efficiency (%) and loading % were calculated to show the relative amount of the Dox in microspheres compared to the initial Dox input in the process. The microspheres were dried at 65 °C overnight in an oven to remove the water. The encapsulation and loading % were calculated with Equations (1) and (2), where *W*_d__, total_, *W*_d__, residual_, and *W*_m_ are the total weight of Dox in the process, residual Dox in the syringe and crosslinking solution, and the total weight of microspheres, respectively.

(1) $$\mathrm{Encapsulation}\;\mathrm{efficiency}\;\left(\%\right)=\frac{W_{d,\;\mathrm{total}}{\;-\;W}_{d,\;\mathrm{residual}}}{W_{d,\;\mathrm{total}}}$$

(2) $$\mathrm{Loading}\;\%=\frac{W_{d,\;\mathrm{total}}{\;-\;W}_{d,\;\mathrm{residual}}}{W_{m}}$$

### 2.5. Characterization of the Calcium Alginate Microspheres

#### 2.5.1. Chemical and Physical Properties

The calcium alginate microspheres were designed to be applied as a hydrogel form. However, to evaluate their chemical and physical properties, microspheres were dried in an oven at 65 °C overnight for the following experiments. The Fourier-transform infrared spectroscopy (FTIR, Tensor 27IR, Bruker, Billerica, MA, USA) was used to obtain infrared spectra of the absorbance of distinct microspheres (before and after cross-linking, without or with NaHCO_3_; without or with Dox). Dried microspheres were finely grounded with KBr (KBr:microshperes = 9:1) to prepare the pellets under the oil pressure of 15 MPa and the infrared spectra were scanned between 400 and 4000 cm^−1^. The microscopic surface features of the calcium alginate microspheres were observed using a scanning electron microscope (SEM, JSM-6380, JEOL Ltd. Tokyo, Japan). Meanwhile, the acetone dispersion method was used to avoid the aggregation of microspheres. A nanoindenter (MST, Nano Indenter^®^ XP, Oak Ridge, TN, USA) was utilized to examine the mechanical properties including Young’s (elastic) modulus (Gpa) and hardness (Gpa). Alternatively, equivalent swelling studies of microspheres in the hydrogel form were performed in pH 7.4 and pH 6.5 PBS at 37 °C. This procedure was repeated until the microspheres reached a constant weight. All samples were prepared in triplicate. The swelling rates were calculated by Equation (3), where *W*_t_ and *W*_i_ are the weight of swelled microspheres and the weight of initial microspheres.

(3) $$\mathrm{Swelling}\;\mathrm{rate}\;\left(\%\right)=\frac{W_{t}{\;-\;W}_{i}}{W_{i}}$$

#### 2.5.2. Analysis of the pH Value in Media and the Drug Release Efficiency In Vitro

A spectrophotometer (NANODROP 2000, ThermoFisher, Waltham, MA, USA) was used to measure Dox release. Since Dox shows a strong absorbance at wavelength 230 (Supplementary Figure S2A), a linear calibration curve was generated by series of dilutions with sterile MiniQ ultrapure water (0.0 to 0.4 mM) (Supplementary Figure S2B). Drug release efficiencies were measured in two solutions, PBS and PBS containing 10% FBS at 37 °C to simulate the medium of cell culture. The microspheres containing NaHCO_3_ and Dox were prepared. All microspheres were washed three times with sterilized and Mini-Q ultrapure water and the water was completely removed by suction. Specifically, 2 × 10^5^ microspheres, calculated by an automated cell counter (TC20, Bio-Rad, Hercules, CA, USA), were placed onto Millicell^®^ hanging cell culture inserts (Merck KGaA, Darmstadt, Germany) in 48-well plates. Afterward, 1.5 mL of PBS or PBS containing 10% FBS were individually added to the plates. Drugs were gradually released from the microspheres. At regular intervals, 1 μL of the supernatant from both the control and the Dox-loaded groups were sampled and analyzed using a NANODROP 2000. The drug release rate in each group was calculated using Equation (4), where *W*_dr_ and *W*_dm_ are the weight of drug release and the total weight of the drug in the microspheres. *W*_dr_ was calculated by OD_230_ using the calibration curve corresponding to the drug concentration and *W*_dm_ was estimated by the weight of 2 × 10^5^ microspheres × the drug loading %. For residual Dox, all solutions were gathered in the CaCl_2_ beaker and measured using the same method.

(4) $$\mathrm{Drug}\;\mathrm{releasing}\;\mathrm{rate}\;\left(\%\right)=\frac{W_{\mathrm{dr}}}{W_{\mathrm{dm}}}$$

### 2.6. In Vitro Anticancer Activities

Two HCC-derived cell lines, Huh-7 and Hep-3B, were used to evaluate the cell viabilities of the Dox-loaded microspheres compared to those without Dox. Cells were maintained in a humidified incubator with 5% CO_2_ at 37 °C with DMEM and MEM containing 10% (*v*/*v*) FBS and 1% (*v*/*v*) antibiotics. Cells (6 × 10^4^) were seeded in a 12-well plate overnight and treated with 5 × 10^4^ microspheres (without or with the Dox) in the cell culture insert to specifically separate cells from the microspheres yet containing sufficient media to cover the microspheres for drug release, as shown in Supplementary Figure S3A. This setup allowed the gradual release of Dox, liberally circulating between the upper and lower chambers. Meanwhile, the wells without cells but with the same microspheres and medium were simultaneously prepared for the subsequent medium replacement to reach the cumulative Dox concentrations. Cells were collected after 4-, 8-, and 12-day treatments and the trypan blue exclusion assay was applied to analyze the cell viability using an automated cell counter (TC20, Bio-Rad, Hercules, CA, USA). Media with the cumulative Dox concentrations were replaced every 4 days to reduce the experimental errors due to the depletion of nutrients in the media. After cell viabilities were determined on days 4 and 8, the remaining unmeasured well plates were replaced with media without cells as shown in Supplementary Figure S3B. Those groups containing microspheres without Dox were subcultured to 6 × 10^4^ cells/well using the same medium. 

### 2.7. Statistics

Statistical analyses were performed using SPSS software (Version 24, IBM, Armonk, NY, USA). One-way analysis of variance (ANOVA) was used to evaluate the significant differences of the cell viabilities among different groups, followed by a Scheffe multiple comparison test. To assess the effects of pH value, multiple regression analysis was applied. The drug release amount and pH value of the medium were the ‘independent variables’ while the cell viability was the ‘dependent variable’. All continuous data are expressed as the means ± SD. For each group, microspheres were prepared with ≥3 repeats. A *p* < 0.05 is considered as statistical significance. 

## 3. Results and Discussion 

### 3.1. The Concentration of CaCl_2_ and Flow Rate Are Critical for a Constant Volume Median Diameter

**Figure 1:**
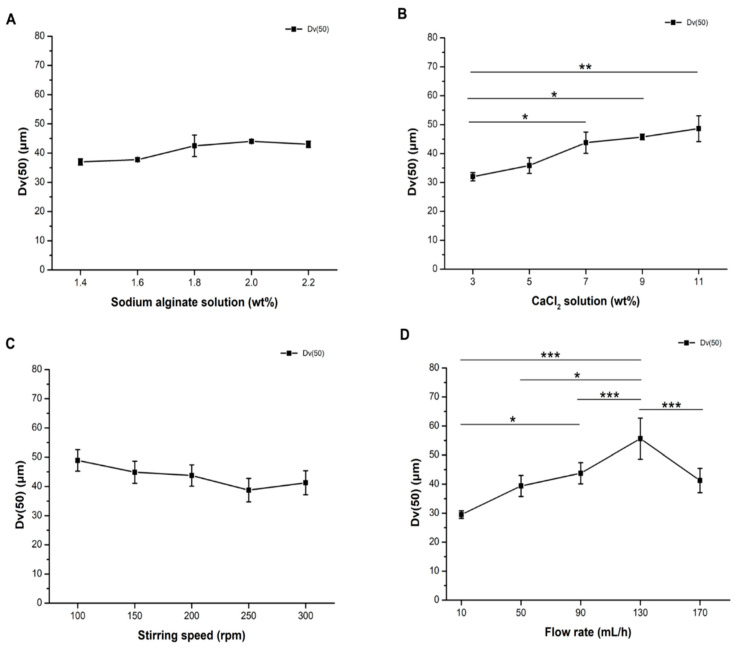
The concentration of CaCl_2_ and flow rate are critical to fabricate alginate microspheres containing Dox with a consistent particle size. In order to obtain the most uniform median particle size of calcium-alginate microspheres, four parameters, (**A**) concentration of sodium alginate (wt%), (**B**) concentration of CaCl_2_ solution (wt%), (**C**) stirring speed (rpm), and (**D**) flow rate (mL/h) of the pump, were screened. One-way ANOVA with post-hoc Scheffe multiple comparison indicated that the concentration of the CaCl_2_ solution and flow rate are the most critical factors among all. Dv(50): Median of diameter volume of the particle size. Statistical significance: * *p* < 0.05, ** *p* < 0.01, *** *p* < 0.001.

Of 17 experimental groups, one-way ANOVA demonstrated the sum of squares (SS), degree of freedom (df), mean of square (MS), *F* test, and *p* value of the Dv(50) between and within groups. No significant difference was found within groups for each parameter while significant differences were identified for the concentration of CaCl_2_ (wt%) and flow rate (Table 1). The Dv(50) in all experiments are shown in Figure 1. Different concentrations of sodium alginate solution (Figure 1A) and stirring speeds (Figure 1C) produced Dox microspheres with Dv(50) of ~37.01 ± 1.09 to 44.03 ± 0.52 μm and ~38.75 ± 4.03 to 49.92 ± 3.65 μm. However, different concentrations of CaCl_2_ and flow rates generated microspheres with Dv(50) of 32.00 ± 1.41 to 48.63 ± 4.48 μm and 29.50 ± 1.32 to 55.63 ± 7.06 μm, respectively. Post-hoc analysis further demonstrated that high CaCl_2_ concentrations (7 to 11 wt%, *p* < 0.05, Figure 1B) and high flow rates of the pump (90 to 130 mL/h, *p* < 0.05, Figure 1D) increased the Dv(50) of microspheres. However, when the flow rate reached 170 mL/h, the Dv(50) of microspheres dropped to a size similar to that of 90 mL/h (*p* < 0.05, Figure 1D). These results suggested that the concentration of CaCl_2_ and flow rate are critical parameters to engender the microspheres with a consistent Dv(50). On the other hand, parameters such as the concentration of sodium alginate solution and the stirring speed did not affect the Dv(50) in the ranges of 1.4 to 2.2 wt% and 100 to 300 rpm. Usually, a low concentration of sodium alginate tends to form microspheres with the smaller particle size after crosslinking with CaCl_2_ [25]. The trend of relatively high sodium alginate concentration resulted in higher Dv(50), consistent with the rationale but without statistical significance. On the other hand, we found that the Dv(50) gradually but non-significantly reduced when the stirring speed increased. This phenomenon can be explained by the fact that high stirring rate causes atomized droplets to quickly exchange along the water flow into the cross-linking liquid at the bottom, reducing the likelihood of collision and fusion of the droplets after dripping and before cross-linking, thus reducing the particle size, which is consistent with earlier studies [26]. 

### 3.2. Optimization of the Flow Rate

**Figure 2:**
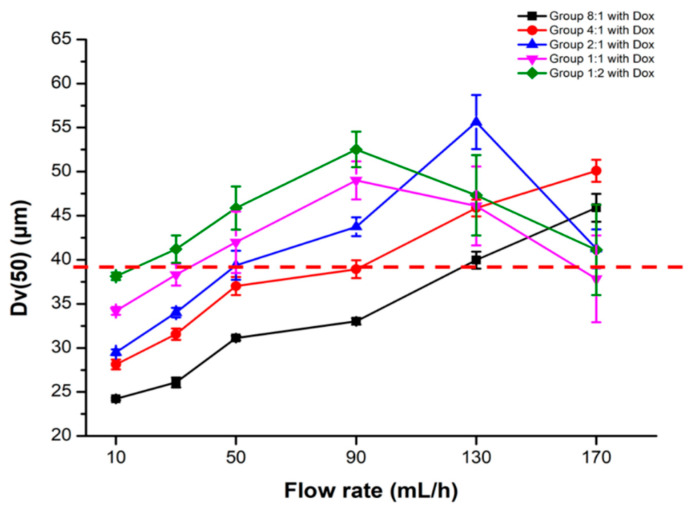
A volume median diameter [Dv(50)] of ~39.0 ± 1.95 μm coexisted in five groups with different alginate:NaHCO_3_ ratios, determining the flow rate in each group for further experiments. Five groups of microspheres with different alginate:NaHCO_3_ ratios were designed.

To identify the optimal flow rate to generate microspheres with a coefficient of variation (CV, SD/mean) of Dv(50) < 5%, five groups with different alginate:NaHCO_3_ ratios were prepared as Groups 8:1, 4:1, 2:1, 1:1, and 1:2, and tested with different flow rates of 10, 30, 50, 90, 130, and 170 mL/h, respectively. The concentration of CaCl_2_ was critical for Dv(50). We first fixed this at the middle value, 7 wt(%), because high CaCl_2_ concentration inclines to form large Dv(50) [27]. The concentration of sodium alginate is not an imperative parameter (Table 1, Figure 1A), therefore 2.2 wt%, the tolerable upper limit of the process, was used across all groups. Besides, there were no significant Dv(50) differences among groups with different stirring speeds, and the speed was fixed at the middle value, 200 rpm (Figure 1C). After screening with different flow rates, Dv(50) ~39 μm with CV < 5% was found to be intersected across five groups (Figure 2), smaller than that (50 to 100 μm) of a commonly used commercial Hepasphere™ (V325HS, Biosphere Medical, Rockland, MA, USA). Accordingly, the correspondent flow rates were 130, 90, 50, 30, and 10 mL/h for Groups 8:1, 4:1, 2:1, 1:1, and 1:2, respectively (Table 2). 

Figure 2 also shows the exact Dv(50) value in each group with a specific flow rate. In all groups, the Dv(50) steady increased as flow rates increase to 90 mL/h. Nevertheless, Dv(50) fluctuated in Groups 2:1 (alginate:NaHCO_3_), 1:1, and 1:2 with relatively high NaHCO_3_ concentrations, when flow rates increased above 90 mL/h. The statistical significances between groups with a specific flow rate are shown in Supplementary Table S4. Briefly, Dv(50) was more diverse between groups at low flow rates (10, 30, 50, and 90 mL/h), while closer between groups at high flow rates (130 and 170 mL/h) with larger standard deviations. As the relative concentrations of NaHCO_3_ increased, CO_2_ gas was accumulated by dissolving Dox (C_27_H_29_NO_11_⋅HCl) and NaHCO_3_ in water and interfered with the process of ion crosslinking of alginate, similar to an earlier report. The aforementioned CO_2_ incorporation also resulted in larger Dv(50) when relative NaHCO_3_ increased. Furthermore, this system effectively increases the Dv(50) at high flow rates, and it also induced droplets by irregular atomization, comparable to previous findings [28].

### 3.3. Dox Encapsulation and Doxloading %

**Figure 3:**
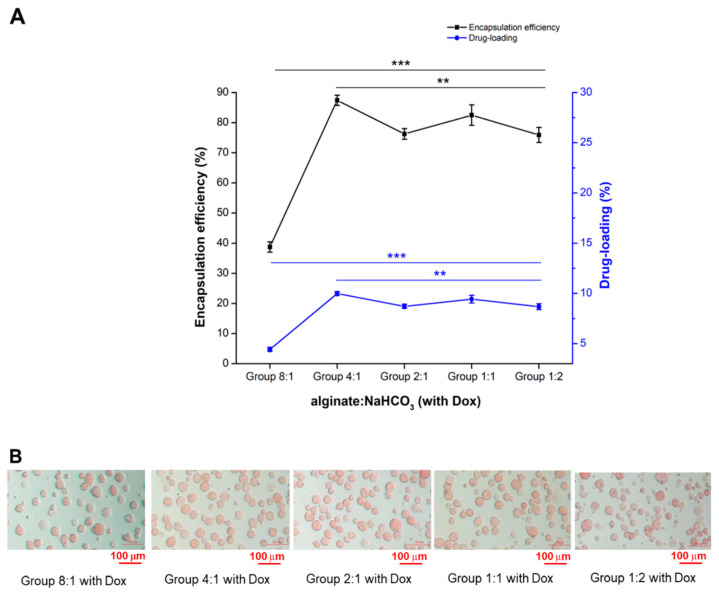
The Dox encapsulation and loading % were similar in three out of five groups with different alginate: NaHCO_3_ ratios. (**A**) Encapsulation and loading %. (**B**) An optical microscope showed the representative morphology of each group. Statistical significance: ** *p* < 0.01, *** *p* < 0.001. Bar scale = 100 μm.

There are two TACE techniques that have been used since 2004, conventional TACE (cTACE) and TACE with drug-eluting beads (DEB-TACE). cTACE includes the intra-arterial injection of a chemotherapeutic drug (e.g., mitomycin C, cisplatin, or Dox), which is emulsified in the oily radio-opaque Lipiodol^®^, followed by intra-arterial injection of an embolic agent (gelatin sponge, polyvinyl alcohol particles, or microspheres). Through cTACE, Lipiodol^®^ carries the drug to the tumor and induces microcirculation embolization [29,30]. Instead, DEB-TACE is non-resorbable embolic microspheres that can be loaded with a specific chemotherapeutic drug for more sustained drug release accompanying embolization [31]. In this study, Dox encapsulation and loading % of microspheres in five groups with different alginate:NaHCO_3_ ratios (8:1, 4:1, 2:1, 1:1, and 1:2) along with its corresponding flow rate (Table 2) are shown in Figure 3A. The averages encapsulation and loading values (%) of Groups 8:1, 4:1, 2:1, 1:1, and 1:2 were estimated as 38.74% ± 1.70, 87.41% ± 1.71, 76.27% ± 1.78, 82.50% ± 3.40, and 75.93% ± 2.51, and 4.43% ± 0.19, 9.99% ± 0.20, 8.72% ± 0.20, 9.43% ± 0.39, and 8.68% ± 0.29, respectively. In general, drug-loading percentages were correlated to the encapsulation efficiencies across all groups. The encapsulation and loading% reached the summit in Group 4:1 (alginate:NaHCO_3_) with a flow rate of 90 mL/h compared to Group 1:2 with a flow rate of 10 mL/h (*p* < 0.01, Figure 3A). Representative images of the appearance of the microspheres from each group are shown in Figure 3B. Although the encapsulation and loading% were similar among Groups 2:1, 1:1, and 1:2, Group 1:2 exhibited fragmented microspheres compared to other groups, which may further affect Dox release, based on our previous study [13]. Fragmented microspheres in relatively high NaHCO_3_ was due to the accumulation of CO_2_ gas by dissolving Dox (C_27_H_29_NO_11_⋅HCl) and NaHCO_3_ in water, resulting in fragile microspheres, comparable to previous reports [2,32]. Moreover, a relatively low NaHCO_3_ concentration along with a high flow rate in Group 8:1 displayed poor encapsulation and loading %. These may be also explained by irregular atomization droplets through high flow rates [28]. Therefore, Groups 4:1, 2:1, and 1:1 (alginate:NaHCO_3_) were subjected to further studies. 

### 3.4. The Chemical and Physical Properties of Alginate Microspheres Containing NaHCO_3_ and Dox

#### 3.4.1. Chemical Properties

**Figure 4:**
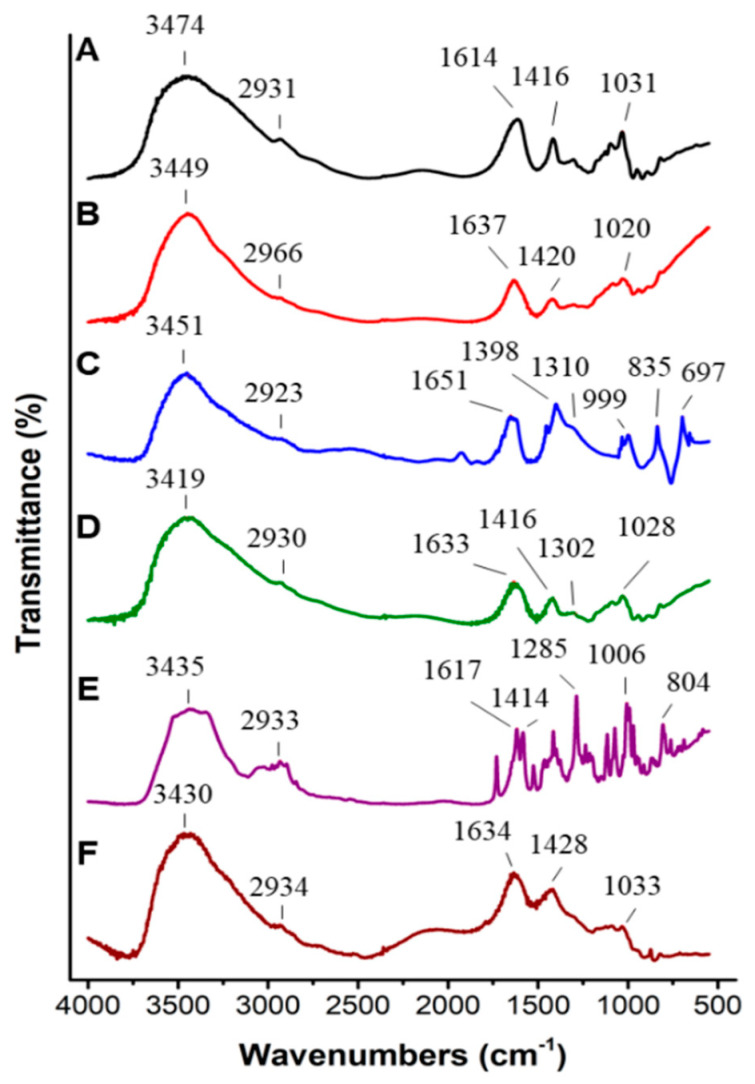
Fourier-transform infrared spectroscopy (FTIR) spectra of calcium alginate microspheres loaded with NaHCO_3_ and Dox. (**A**) Sodium alginate power. (**B**) Calcium alginate microspheres. (**C**) NaHCO_3_ powder. (**D**) Calcium alginate microspheres containing NaHCO_3_ (B + C). (**E**) Dox. (**F**) Calcium alginate microspheres containing NaHCO_3_ and Dox (D + E). Peaks shifting suggest Dox was successfully incorporated into calcium alginate microspheres containing NaHCO_3_. Overall, peaks at specific regions (wavenumbers, cm^−1^) are 3419-3474: hydroxyl (O-H) and 2923-2966: alkyl group (C-H) stretching vibrations. Peaks at 1614 and 1416 (A), 1637 and 1420 (B), 1633, 1416 (D), 1634 and 1428 (F) indicate carboxylate salt (C=O) symmetrical and asymmetrical vibrations; 1031 (A), 1020 (B), 1028 (D), and 1033 (F) show C-O stretching vibrations. Peaks at 835 and 697 (C) display the structure characteristics of carbonate; 1310 and 999 (C) are the in-plane and out-of-plane bending vibrations of C-O-H, respectively. For Dox (E), 1617: amines (N-H), 1414: aromatic, 1285, and 804: C-H stretching vibrations.

**Figure 5:**
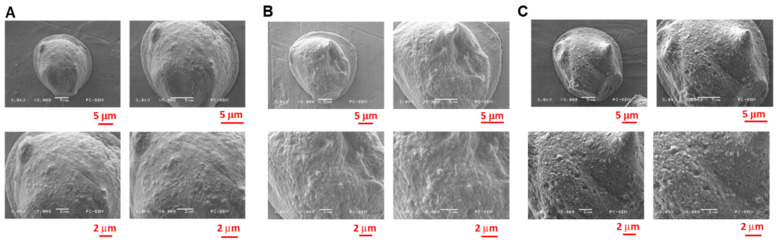
The surface microstructures of calcium alginate microspheres with different NaHCO_3_ concentrations. A scanning electron microscope showed the surfaces of fabricated microspheres at 3000×, 5000×, 7000×, and 9000× magnification. (**A**) Group 4:1 (Alginate:NaHCO_3_), (**B**) Group 2:1, and (**C**) Group 1:1.

Alterations of the chemical structure of sodium alginate and calcium alginate microspheres after crosslinking with NaHCO_3_ were studied by an FTIR spectrum (Figure 4). Shifting of peaks (wavenumbers) were observed in calcium alginate microspheres by calcium crosslinking (Figure 4B) compared to sodium alginate power (Figure 4A), in calcium alginate microspheres containing NaHCO_3_ through NaHCO_3_ incorporation (Figure 4D) compared to calcium alginate microspheres (Figure 4B) and NaHCO_3_ powder (Figure 4C), and in calcium alginate microspheres containing NaHCO_3_ and Dox via Dox encapsulation (Dox-NaHCO_3_, Figure 5F) compared to calcium alginate microspheres containing NaHCO_3_ only (Figure 4D). This profile suggested that Dox-NaHCO_3_ microspheres were step-by-step fabricated successfully.

#### 3.4.2. Physical Properties

The surface microstructures of Dox-NaHCO_3_ microspheres were observed using an SEM. Calcium alginate microspheres with Dox and different relative NaHCO_3_ concentrations, Groups 4:1 (alginate:NaHCO_3_), 2:1, and 1:1, are shown in Figure 5 with different magnifications, whereby 3000×, 5000×, 7000×, and 9000× SEM identified that these microspheres shrunk due to the drying process and became irregular on their surfaces. Much smoother surfaces were found in Group 4:1 with a relatively low NaHCO_3_ concentration (Figure 5A) compared to Group 2:1 (Figure 5B) and Group 1:1 (Figure 5C) since less CO_2_ gas was accumulated when NaHCO_3_ mixed with Dox in the process. Under 3000× magnification, the average diameter of Group 1:1 was larger compared to those of Groups 2:1 and 4:1, potentially due to the fact that low relative alginates in Group 1:1 were subjected to be dehydrated compared to Group 2:1 and 4:1. 

**Figure 6:**
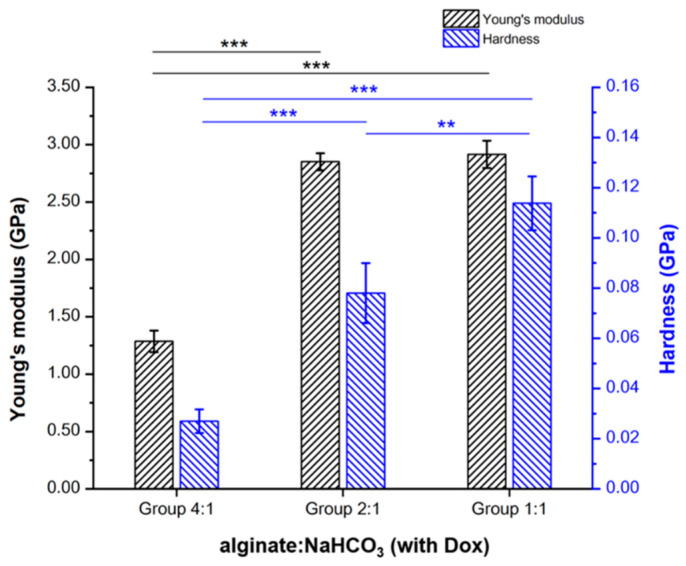
Microspheres with relatively high NaHCO_3_ concentrations resulted in higher Young’s modulus and hardness. Among three groups with different alginate:NaHCO_3_ (Groups 4:1, 2:1, and 1:1), a nanoindenter identified their respective Young’s modulus and hardness. Statistical significance: ** *p* < 0.01, *** *p* < 0.001.

Among three groups with different alginate:NaHCO_3_ ratios, Young’s modulus (GPa) was similar between Groups 2:1 (2.85 ± 0.07) and 1:1 (2.92 ± 0.12), and higher than that of 4:1 (1.29 ± 0.09) (*p* < 0.001). Likewise, the hardness (GPa) was highest in Group 1:1 (0.11 ± 0.01) compared to those of 2:1 (0.08 ± 0.01; *p* < 0.01) and 4:1 (0.03 ± 0.00; *p* < 0.001). In addition, the hardness of Group 2:1 was higher than that of Group 4:1 (*p* < 0.001) (Figure 6). These observations suggested that Group 1:1 retains both elasticity (to resist deformation) and hardness. Thus, in the presence of the same amount of Dox, the relative NaHCO_3_ concentration is a critical factor to determine the elasticity and hardness (surface-to-mass ratio) of the alginate microspheres. 

#### 3.4.3. Swelling Ratios of the Dox-NaHCO_3_ Alginate Microspheres in PBS with pH 7.4 and pH 6.5

**Figure 7:**
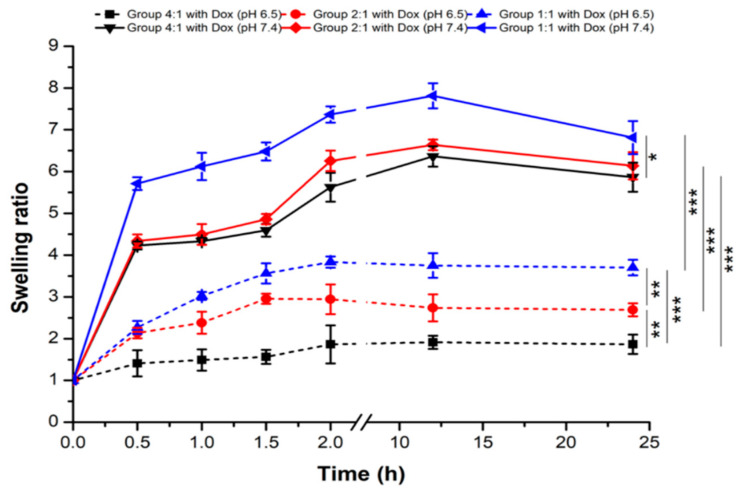
Higher swelling ratios in Dox-loaded microspheres in pH 7.4 PBS compared to pH 6.5 PBS across all groups. Three different groups of microspheres with different alginate:NaHCO_3_ were 4:1, 2:1, and 1:1, and swelling rates were measured by the weighting difference. Statistical significance: * *p* < 0.05, ** *p* < 0.01, *** *p* < 0.001.

We prepared two PBS solutions with pH 7.4 (normal body fluids) and pH 6.5 (to mimic the tumor microenvironment), respectively, to measure the swelling rates of Dox-NaHCO_3_ alginate microspheres at their hydrogel states from 0 to 24 h. After microspheres were immersed in PBS, the swelling ratios were rapidly increased during the first 30 min and gradually increased afterward in all groups (alginate:NaHCO_3_ = 4:1, 2:1, and 1:1, respectively, Figure 7). Swelling ratios can be clustered into two groups: pH 7.4 and pH 6.5 at 24 h after immersion in PBS. In general, microspheres with relatively high NaHCO_3_ concentrations showed higher swelling ratios in the same medium. The swelling ratios of microspheres were much higher in pH 7.4 compared to those with pH 6.5 in Group 4:1 (4.23 ± 0.09 to 6.36 ± 0.25 vs. 1.41 ± 0.32 to 1.91 ± 0.16; *p* < 0.001), 2:1 (4.34 ± 0.16 to 6.64 ± 0.12 vs. 2.13 ± 0.13 to 2.96 ± 0.12; *p* < 0.001), and 1:1 (5.71 ± 0.15 to 7.81 ± 0.30 vs. 2.27 ± 0.15 to 3.83 ± 0.13; *p* < 0.001) from 0.5 h to 24 h (Supplementary Table S5). These observations, consistent with an earlier report, were due to the acidic condition that enhanced COOH formation and compacted the microsphere, while the alkaline condition, on the other hand, promoted Ca^2+^ binding to two COO- groups and relaxed the structure of microspheres [33]. At pH 6.5, the swelling rates were different in Group 4:1 (alginate:NaHCO_3_) vs. 2:1 (*p* < 0.01), Group 4:1 vs. 1:1 (*p* < 0.001), and Group 2:1 vs. 1:1 (*p* < 0.01) after immersion into PBS for 0.5 to 24 h. Instead, at pH 7.4, due to similar swelling ratios between Group 4:1 and 2:1, higher swelling ratios were found in Group 4:1 vs. 1:1 (*p* < 0.05) and 2:1 vs. 1:1 (*p* < 0.01) after immersion in PBS for 0.5 h to 12 h (Supplementary Table S6). However, the significant difference was only retained in Group 4:1 vs. 1:1 after immersion in PBS for 24 h owing to the degradation (loss of weight and fragmentation) of the microspheres, resulting in large standard deviations (Figure 7). Accordingly, in pH 7.4 PBS, the microspheres absorbed PBS rapidly, and the swelling ratios reached up to ~7.37 ± 0.19-fold (Group 1:1) compared to ~3.83 ± 0.13-fold (Group 1:1) at 2 h in pH 6.5 PBS. The average swelling rate is larger than those of Hepaspheres™ in pH 7.4 PBS (~4 folds, 200 to 400 μm). Therefore, after the multiplication of its original size, Dv(50)~39 μm, Group 1 microspheres in pH 7.4 PBS can be expanded to ~287 μm. This Dv(50) falls into the diameter range of Hepaspheres™ after expansion, reinforcing that Dox-NaHCO_3_ microspheres fabricated in this study are suitable for clinical usage. 

Indeed, the sensitivity to pH value is one important feature of the calcium alginate microspheres. The swelling ratios were markedly suppressed under a low pH environment. It has been reported that alginate-poly(γ-glutamic acid) composite microparticles could uptake hundreds of times of their weight in water. Both the maximum water uptake ratio and the swelling rate were increased by enlarging the amount of poly(γ-glutamic acid) in the composite [34]. Through the immersion of quercetin/chitosan/sodium alginate microspheres in PBS with three different pH values, simulated gastric fluid pH 1.2, and simulated intestinal fluid pH 6.8 and 7.4, the swelling index decreased as the pH value declined [35,36]. This phenomenon was potentially caused by acid conditions that protonated the carboxylate groups of the polymers on the surface of microspheres. Thus, the formation of hydrogen bonds with insoluble alginate in a low pH fluid increases the structure stability, hampering the diffusion of additional fluid into the core of each microsphere [36], consistent with the observations in our previous [13] and this study. 

### 3.5. Relatively High NaHCO_3_ Concentrations and 10% FBS Enhance Accumulated Dox Release Rates in PBS

**Figure 8:**
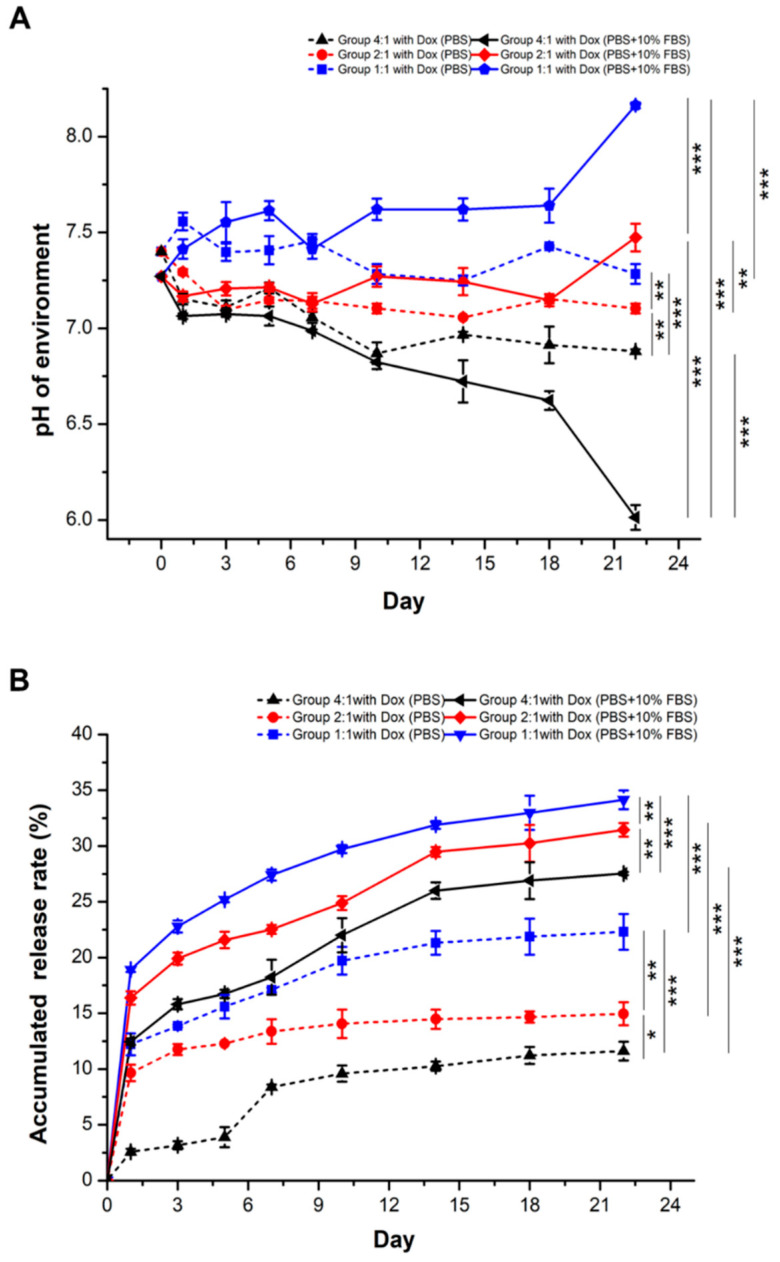
Alterations of the environmental pH values and Dox accumulated release rates after the immersion of the Dox-and NaHCO_3_-loaded microspheres in pH 7.4 PBS and pH 6.5 PBS for 22 days. (**A**) A pH meter was used to measure the pH value. (**B**) Accumulated Dox release rates were measured using a NanoDrop spectrophotometer in different groups. Statistical significance: * *p* < 0.05, ** *p* < 0.01, *** *p* < 0.001.

The objective of adding 10% FBS to PBS was to simulate an environment of cell culture. The pH values in the media were measured in Figure 8A. In Group 4:1, 2:1, and 1:1 (alginate:NaHCO_3_), the average pH values ranged from 6.87 ± 0.06 to 7.56 ± 0.05 in PBS while the average pH values ranged from 6.01 ± 0.06 to 8.16 ± 0.02 in PBS containing 10% FBS. Overall, the distribution of average pH values for Groups 4:1, 2:1, and 1:1 were more centralized in PBS compared to those in PBS containing 10% FBS. From day 1 to day 22 after immersion, the pH values oscillated to some extent from microspheres across all groups in PBS as well as in PBS containing 10% FBS. At day 22, the average pH values in each group (4:1, 2:1, and 1:1, respectively) were different in PBS containing 10% FBS compared to those in PBS only (*p* < 0.01 to *p* < 0.001, Supplementary Table S7). The average pH values in Group 4:1 were always lower than those in Groups 2:1 and 1:1, containing relatively higher NaHCO_3_ concentrations in PBS and PBS with 10% FBS (*p* < 0.01, Figure 8A, Supplementary Table S8). In PBS containing 10% FBS, the average pH values suddenly increased in Groups 1:1 and 2:1 and abruptly decreased in Group 4:1 at day 22 compared to day 18, due to the disruption of microspheres. Indeed, many unknown components of FBS might be the cause of the degradation of microspheres. The relative ratios of NaHCO_3_ to alginate in microspheres increased and decreased the pH values. Accordingly, in both PBS and PBS containing 10% FBS, Groups 2:1 and 1:1 with relatively high NaHCO_3_ concentrations maintained better physiological pH values (~pH 7.4) compared to those of Group 4:1 with relative low NaHCO_3_ concentrations. 

Accumulated Dox release rates are shown in Figure 8B. In all groups, Dox was released rapidly at day 1 and gradually released thereafter in PBS as well as PBS containing 10% FBS. Accumulated Dox release rates remained similar starting day 14 to day 22, reaching a plateau in each group. Dox release in PBS increased every 2–3 days in Groups 4:1 (2.59% ± 0.27 to 11.61% ± 0.84), 2:1 (9.66% ± 0.74 to 14.66% ± 1.04) and 1:1 (12.22% ± 0.99 to 22.31% ± 1.61). Likewise, Dox release in PBS containing 10% FBS increased every 2–3 days in Groups 4:1 (12.46% ± 0.28 to 27.53% ± 0.14), 2:1 (16.37% ± 0.60 to 31.46% ± 0.61), and 1:1 (18.92% ± 0.22 to 34.14% ± 0.84) (Figure 8B), i.e., accumulated release rates were higher in PBS containing 10% FBS compared to those in PBS only across all groups (4:1, 2:1, and 1:1, *p* < 0.001, Figure 8B; Supplementary Table S9), similar to our previous study [13]. FBS contains proteins/enzymes and unknown factors and destabilized the structure of alginate microspheres, potentially lead to faster drug release. At day 22, Dox release rates in Group 4:1 vs. 2:1 (*p* = 0.04, *p* = 0.001), 4:1 vs. 1:1 (*p* < 0.001, *p* = 0.000), and 2:1 vs. 1:1 (*p* = 0.005, *p* = 0.005) were different in PBS and PBS containing 10% FBS, respectively (Supplementary Table S10), i.e., higher relative NaHCO_3_ concentrations resulted in more Dox releases. Low environmental pH comprises a high concentration of hydrogen ions binding to the carboxylic acid group in alginates, provoking further compacts of the microspheres to prevent swelling and drug-releasing, consistent with earlier studies [33]. These aspects were different from another study, which showed that low pH value favored Dox release from fullerene [37], perhaps due to the nature of these materials are distinct. In addition, Jagusiak et al. (2020) designed a pH-sensitive triple complex containing single-walled carbon nanotubes, Congo red, and Dox for controlled release of Dox. The decrease in pH changed the structure and stability and ensured efficient drug release. Unfortunately, Congo red is not a biocompatible compound [38]. In this study, both FBS in the environmental media and relatively high NaHCO_3_ concentrations in alginate microspheres enhance Dox release, reinforcing that these alginate microspheres are suitable for drug delivery in vivo. Further, compared to the most popular material, HapaShpere™, the release of Dox was observed as early as 2 h after TACE, reached the peak at 3 days, and remained at measurable levels up to 7 days [39], and our Dox-NaHCO_3_ alginate microspheres exhibited notably sustained release to at least 14 days after treatments.

### 3.6. Alginate Microspheres Containing Relatively High NaHCO_3_ Concentrations Strongly Inhibit Cancer Cell Viabilities In Vitro

**Figure 9:**
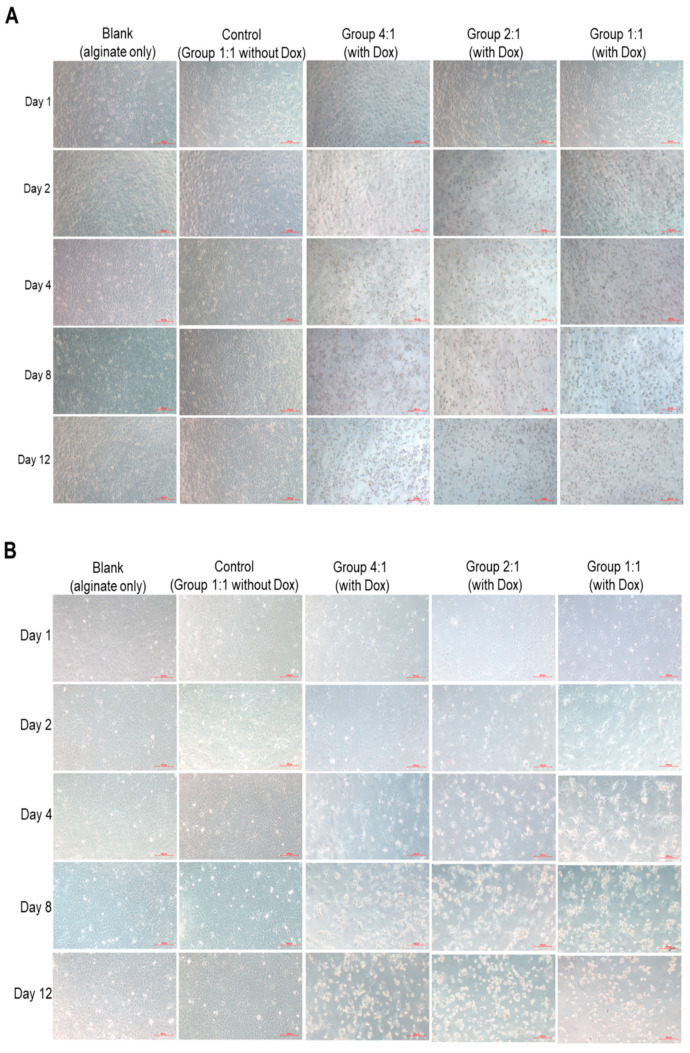

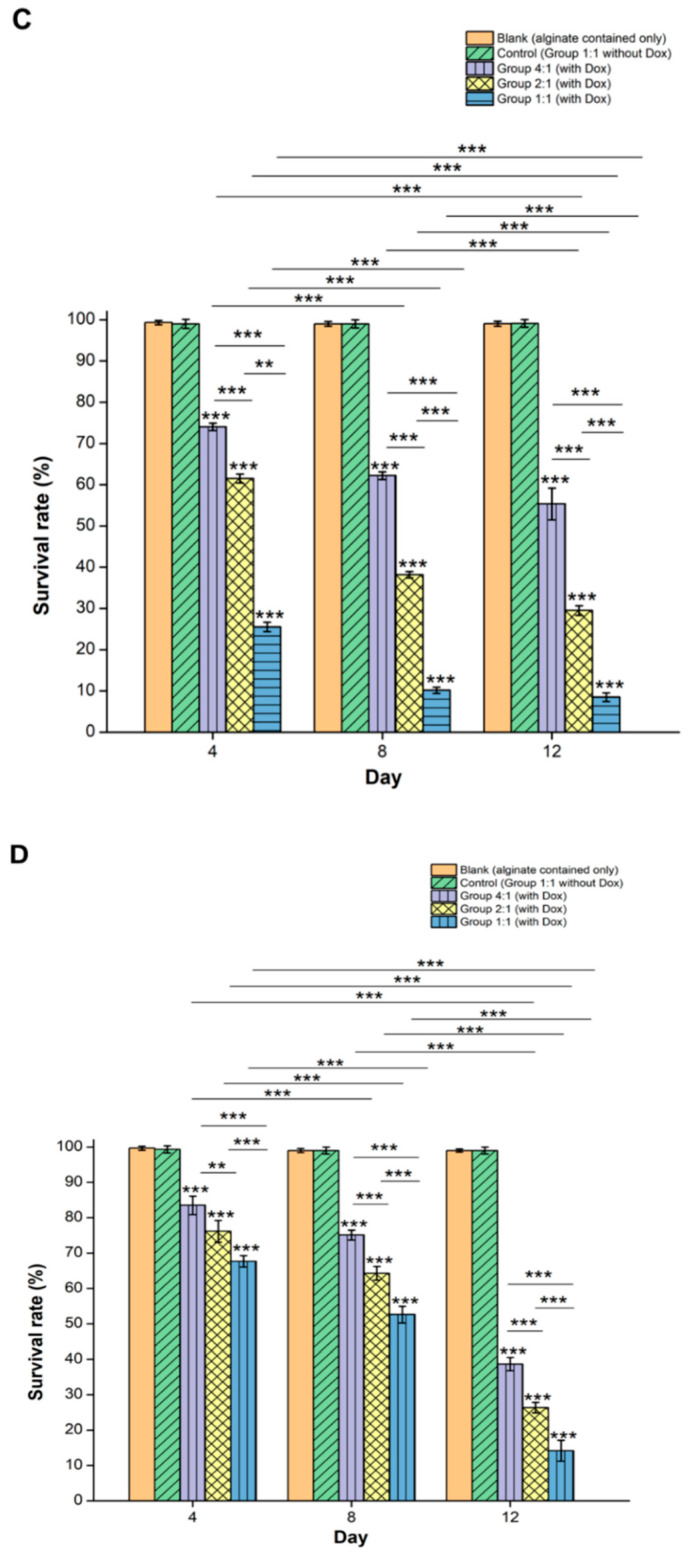
Treatments of Dox microspheres with relatively high NaHCO_3_ concentrations significantly reduced cell viabilities in two distinct HCC derived-cells, Huh-7 and Hep-3B, in vitro. An inverted microscope shows cells of (**A**) Huh-7 cells and (**B**) Hep-3B cells before and after treatments with blank (alginate only), control [Group 1:1 (alginate:NaHCO_3_) without Dox, Group 4:1 with Dox, 2:1 with Dox, and 1:1 with Dox, respectively, after treatments for 1, 2, 4, 8, and 12 day, scale bar = 200 μm. (**C**,**D**) In both cell lines, Dox microspheres with relative high NaHCO_3_ concentrations steadily reduced cell viabilities at day 4, 8, and 12 after treatments compared to the control group. Within the same group, prolonged treatments from day 4 to day 12 decreased cell viabilities as well. Statistical significance: ** *p* < 0.01, *** *p* < 0.001.

Two hepatocellular carcinomas-derived cell lines, Huh-7 [*Tumor Protein 53* (*TP53*)-positive)] and Hep-3B (*TP53*-deficient) with distinct genetic backgrounds, were used to evaluate the cytotoxicity of alginate microspheres with or without NaHCO_3_ and/or with or without Dox. *TP53* is a tumor suppressor gene, the encoded protein responds to diverse cellular stresses to regulate the expression of target genes, thereby inducing cell cycle arrest, apoptosis, senescence, DNA repair, or changes in metabolism [40]. To perform the experiments in cells with distinct genetic backgrounds would provide more general results. A total of five groups were designed: Alginate microspheres (blank), alginate microspheres with NaHCO_3_ (1:1) without Dox (control), Group 1:1 (alginate:NaHCO_3_) with Dox, Group 2:1 with Dox, and Group 4:1 with Dox. The cell morphologies of both cell lines were not changed after treatments with alginate microspheres (blank) from day 1 to day 12, while the cell densities were gradually increased with incubation time. Similar aspects were observed in the control group (alginate:NaHCO_3_ = 1:1 without Dox). However, cell shrinkage was observed starting day 2 to day 12 in Group 4:1, 2:1, and 1:1 with Dox in Huh-7 (Figure 9A) and Hep-3B (Figure 9B) cells. 

In both cell lines, the cell viabilities were quite stable between blank (Huh-7: 99.32% ± 0.50 to 99.03% ± 0.59; Hep-3B: 99.67% ± 0.58 to 99.00 ± 0.47) and control (Huh-7: 99.00% ± 1.09 to 99.10% ± 0.92; Hep-3B: 99.33% ± 1.04 to 99.00% ± 0.99) after treatments with different microspheres for 4 to 12 days. Cell viabilities were stepwise decreased in Group 4:1 (Huh-7: 74.02% ± 0.89 to 55.33% ± 3.83, *p* < 0.001; Hep-3B: 83.50% ± 2.59 to 38.67% ± 1.86, *p* < 0.001), 2:1 (Huh-7: 61.50% ± 1.04 to 29.51% ± 1.15, *p* < 0.01; Hep-3B: 76.17% ± 3.06 to 26.36% ± 1.47, *p* < 0.001), and 1:1 (Huh-7: 25.50% ± 1.13 to 8.51% ± 1.05, *p* < 0.001; Hep-3B: 67.65% ± 1.63 to 14.14% ± 2.96, *p* < 0.001) compared to those of blank and control after treatments for 4 to 12 days. Significant differences were observed in pairwise comparisons between every two groups after treatments for 4 days (*p* < 0.01), 8 days (*p* < 0.001) and 12 days (*p* < 0.001). Cell viabilities were likewise stepwise decreased within the same group after treatments for 12 days compared to those of 8 days (*p* < 0.001) and 4 days (*p* < 0.001), respectively, and 8 days compared to that of 4 days (*p* < 0.001) (Figure 9C). Similar tendencies were found in Hep-3B cells, except for the decrease trends of cell viabilities were milder compared to those of Huh-7 cells (Figure 9D), owing to Hep-3B being a *TP53*-deficient cell line [41]. Resistance to TP53-mediated growth arrest and apoptosis in Hep-3B cells has been reported [42]. One limitation of this experiment was that no media can last for 12 days in cell culture due to the metabolites released by the cells. During treatments, cells may uptake Dox, and the replacement of the media with cells by media without cells may introduce extra Dox. However, the extra Dox was assumed to be the same among groups because we used the same cell number in each group. Furthermore, we measured the relative cell viabilities compared to the control in each group, which calibrates the effects of extra Dox. Multiple linear regression analysis with the model summary was next performed to evaluate whether Dox and/or pH affect the cell viabilities (Table 3). Supplementary Table S11 lists the model summary. In Huh-7 and Hep-3B cells, the R^2^ values ranged from 0.723 to 0.975 and the adjusted R^2^ values ranged from 0.686 to 0.972, across days 4, 8, and 12 after treatments with Dox-NaHCO_3_-microspheres. These estimates indicated that the variable, i.e., the cell viability, can be highly explained by two independent variables, the Dox release amount and the environmental pH value. ANOVA next showed the Dox release amount and the environmental pH impact on cell viabilities in both cell lines after treatments with Dox-NaHCO_3_-microspheres for 4 days (*p* < 0.001), 8 days (*p* < 0.001), and 12 days (*p* < 0.001) (Supplementary Table S12). Multiple linear regression analysis showed negative standardized coefficients between the cell viabilities and the environmental pH as well as Dox releases after treatments with Dox-NaHCO_3_-calcium alginate microspheres for 4, 8, and, 12 days in both cell lines. Although Dox release slowed down after treatments with Dox-NaHCO_3_-microspheres from day 4 to day 12 (*p* = 0.025 to *p* = 0.874), the environmental pH value continued to play an important role to inhibit cell viabilities (*p* = 0.000 to *p* = 0.007) in both cell lines (Table 3). 

Compared to current commercially available TACE materials such as DC Bead™ and HapaSphere™, which are not biodegradable, alginate-NaHCO_3_ microspheres fabricated in this study offer biodegradable advantages for re-TACE in local recurrence patients. Biodegradable alginate-NaHCO_3_ microspheres can avoid the occlusion of arteries for patients who meet the criteria for liver transplantation. For the first time, we incorporated NaHCO_3_ into the biodegradable microspheres for TACE to notably counteract the tumor microenvironment and provide significant and better cytotoxicities for HCC-derived cells. 

## 4. Conclusions

Taken together, ultrasonic atomization was used to fabricate alginate microspheres. The concentration of CaCl_2_ and flow rate of the pump are critical parameters to produce calcium microspheres with an average Dv(50)~39 μm. Among several groups with different alginate:NaHCO_3_ ratios, flow rates were optimized in each group. Dox-loading percentages were correlated to encapsulation efficiencies across five groups with different alginate:NaHCO_3_ ratios. Fourier spectrum showed the successful fabrication of alginate microspheres containing Dox and NaHCO_3_, step-by-step. The surfaces are rough and porous in microspheres with higher NaHCO_3_ concentrations. Young’s modulus and hardness reach the highest value in Group 1:1 (alginate:NaHCO_3_). In vitro, swelling rates are higher across three groups with different alginate:NaHCO_3_ ratios in pH 7.4 PBS compared to pH 6.5 PBS. Alginate microspheres containing Dox and relatively high NaHCO_3_ concentrations showed higher environmental pH values, Dox release rates in PBS and PBS containing 10% FBS, and significantly inhibited cell viabilities in two HCC-derived cells, Huh-7 and Hep-3B, in vitro compared to those containing relative low NaHCO_3_ concentrations. Incorporating NaHCO_3_ in calcium alginate microspheres with Dox significantly decreased cell viabilities in distinct HCC-derived cells. 

## Figures and Tables

**Table 1:** The concentration of CaCl_2_ and flow rate significantly affected the volume median diameter of calcium alginate microspheres.

One-Way ANOVA						
Parameters/Variable		SS	df	MS	F	*p* Value
Sodium alginate solution (wt%)	Between Groups	153.077	4	38.269	0.893	0.473
	Within Groups	3084.728	72	42.843		
	Total	3237.805	76			
CaCl_2_ solution (wt%)	Between Groups	800.926	4	200.232	5.916	0.000
	Within Groups	2436.879	72	33.846		
	Total	3237.805	76			
Stirring speed (rpm)	Between Groups	372.172	4	93.043	2.338	0.063
	Within Groups	2865.633	72	39.800		
	Total	3237.805	76			
Flow rate (mL/h)	Between Groups	1193.659	6	198.943	6.813	0.000
	Within Groups	2044.146	70	29.202		
	Total	3237.805	76			

SS: Sum of squares, df: Degree of freedom, MS: Mean square, F: F test.

**Table 2:** Optimization of the flow rate in five groups with different alginate:NaHCO_3_ ratios to fabricate the microspheres with a consistent volume median diameter (~39 μm).

Alginate: NaHCO_3_ (wt%:wt%)	Sodium Alginate Solution (wt%)	CaCl_2_ Solution (wt%)	Stirring Speed (rpm)	^a^ Optimized Flow Rate (mL/h)
8:1	2.2	7	200	130
4:1				90
2:1				50
1:1				30
1:2				10

^a^ Flow rates: 10, 30, 50, 90, 130 and 170 mL/h, respectively, were tested.

**Table 3:** Multiple linear regression analysis demonstrated that high environmental pH values reduced the cell viabilities after treatments with Dox-NaHCO_3_-/calcium alginate microspheres for 4, 8, and 12 days.

Cells		Unstandardized Coefficient	Standardized Coefficient	*t* Test	*p* Value	VIF
Huh-7						
Day 4	Constant	1989.646		2.949	0.010	
	pH	−22.939	−0.531	−3.152	0.007	1.535
	Dox	−14551.143	−0.421	−2.499	0.025	1.535
Day 8	Constant	504.923		0.767	0.455	
	pH	−46.177	−0.938	−8.664	0.000	1.861
	Dox	−1043.472	−0.020	−0.185	0.856	1.861
Day 12	Constant	−104.376		−0.405	0.691	
	pH	−18.435	−1.022	−14.526	0.000	1.778
	Dox	−2076.344	−0.067	−0.956	0.354	1.778
**Huh-3B**						
Day 4	Constant	1309.960		2.630	0.019	
	pH	−25.373	−0.686	−4.723	0.000	1.535
	Dox	−8687.680	−0.293	−2.021	0.062	1.535
Day 8	Constant	343.403		1.480	0.160	
	pH	−33.141	−0.981	−17.648	0.000	1.861
	Dox	−319.867	−0.009	−0.161	0.874	1.861
Day 12	Constant	515.078		0.902	0.381	
	pH	−21.376	−0.894	−7.606	0.000	1.778
	Dox	−2753.621	−0.067	−0.572	0.576	1.778

Dox: Dox release amount (μg), VIF: Variance inflation factor.

## Data Availability

Not applicable.

## Supplementary Materials

The following are available online at https://www.mdpi.com/article/10.3390/pharmaceutics13091417/s1, Table S1: A 4-by-5 experiment was designed to identify the optimal combination(s) for the fabrication of calcium alginate microspheres. Table S2: Codes and levels of all parameters. Table S3: A total of 17 experiments were performed to detect the optimal parameters. Table S4: Comparison of the volume median diameters (Dv(50)) between different groups (alginate:NaHCO_3_) with a specific flow rate. Table S5: Comparison of the swelling ratios between microspheres after the immersion into pH 6.5 PBS and pH 7.4 PBS from 0.5 to 24 h within each group with a specific alginate:CaCl_2_ ratio. Table S6: Comparison of the swelling rates of microspheres between groups (with different alginate:NaHCO3 ratios) after the immersion into pH 6.5 PBS and pH 7.4 PBS from 0.5 to 24 h. Table S7: Comparison of the average pH values between microsphere in PBS and PBS containing 10% FBS in different groups with different alginate:NaHCO_3_ ratios after the immersion from day 1 to day 22. Table S8: Comparison of the average pH values among microspheres with different alginate:NaHCO_3_ ratios in PBS and PBS containing 10% FBS after the immersion from day 1 to day 22. Table S9: Comparison of the accumulated Dox release rates between microspheres in PBS and PBS containing 10% FBS in different groups (ratio = alginate:NaHCO_3_) from day 1 to day 22 after immersion. Table S10: Comparison of the accumulated Dox release rates among microspheres in PBS and PBS containing 10% FBS (ratio = alginate:NaHCO_3_) from day 1 to day 22. Table S11: Model summary of multiple linear regression analysis used to evaluate the effects of Dox release amount (μg) and the environmental pH value on cell viabilities. Table S12: Analysis of variance (ANOVA) showed the regression, residual, F, and *p* values after treatments with Dox-NaHCO_3_ calcium alginate microspheres for 4, 8, and 12 days. Figure S1: The experimental setup where the mixture (sodium alginate, NaHCO3 and/ir Dox) was located in a syringe and the syringe was squeezed by an infusion pump, Figure S2: A calibration curve was generated to detect Dox concentration, Figure S3: Media with the cumulative Dox concentrations were replaced every 4 days to reduce the experimental errors due to the depletion of nutrients in the media.
