# Supplementary material for: Sustained-Release and pH-Adjusted Alginate Microspheres-Encapsulated Doxorubicin Inhibit the Viabilities in Hepatocellular Carcinoma-Derived Cells
Source: Pharmaceutics. 2021 Sep 7;13(9):1417. doi: 10.3390/pharmaceutics13091417 (PMC8471522; doi:10.3390/pharmaceutics13091417)
Supplement: Supplementary file 1 [file pharmaceutics-13-01417-s001.zip › pharmaceutics-1337412-supplementary.pdf]

pharmaceutics

Supplementary Materials

Sustained-Release and pH-Adjusted Alginate Microspheres-Encapsulated Doxorubicin Inhibit the Viabilities in Hepatocellular Carcinoma-Derived Cells

Cheng-Tang Pan, Ruei-Siang Yu, Chih-Jung Yang, Lih-Ren Chen, Zhi-Hong Wen, Nai-Yu Chen, Hsin-You Ou, Chun-Yen Yu and Yow-Ling Shiue

Table S1. A 4 by 5 experiment was designed to identify the optimal combination(s) for the fabrication of calcium alginate microspheres.

Code	Parameter	Level	Unit
A	Concentration of sodium alginate	1.4, 1.6, 1.8, 2.0, 2.2	wt%
B	Concentration $CaCl_2$	3, 5, 7, 9, 11	wt%
C	Stirring speed	100, 150, 200, 250, 300	rpm
D	Flow rate	10, 50, 90, 130, 170	mL/h

Table S2. Codes and levels of all parameters.

Level/Code	A (wt%)	B (wt%)	C (rpm)	D (mL/h)
1	1.4	3	100	10
2	1.6	5	150	50
3	1.8	7	200	90
4	2.0	9	250	130
5	2.2	11	300	170

A: Sodium alginate solution, **B:** $CaCl_2$ solution, **C:** Stirring speed, **D:** Flow rate of the pump.

Table S3. A total of 17 experiments were performed to detect the optimal parameters.

Experiment	A	B	C	D
[a]1	A3	B3	C3	D3
2	**A1**	B3	C3	D3
3	**A2**	B3	C3	D3
4	**A4**	B3	C3	D3
5	**A5**	B3	C3	D3
6	A3	**B1**	C3	D3
7	A3	**B2**	C3	D3
8	A3	**B4**	C3	D3
9	A3	**B5**	C3	D3
10	A3	B3	**C1**	D3
11	A3	B3	**C2**	D3
12	A3	B3	**C4**	D3
13	A3	B3	**C5**	D3
14	A3	B3	C3	**D1**
15	A3	B3	C3	**D2**
16	A3	B3	C3	**D4**
17	A3	B3	C3	**D5**

[a] Experiment 1 served as the reference group.

Table S4. Comparison of the volume median diameters [Dv(50)] between different groups (alginate:$NaHCO_3$) with a specific flow rate.

	p Value (ANOVA/Scheffe Test)									
**Flow Rate**	**8:1 vs. 4:1**	**8:1 vs. 2:1**	**8:1 vs. 1:1**	**8:1 vs. 1:2**	**4:1 vs. 2:1**	**4:1 vs. 1:1**	**4:1 vs. 1:2**	**2:1 vs. 1:1**	**2:1 vs. 1:2**	**1:1 vs. 1:2**
10	0.000	0.000	0.000	0.000	0.016	0.000	0.000	0.000	0.000	0.000
30	0.000	0.000	0.000	0.000	0.273	0.000	0.000	0.010	0.000	0.054
50	0.068	0.010	0.001	0.000	0.749	0.151	0.006	0.679	0.041	0.313
90	0.009	0.000	0.000	0.000	0.038	0.000	0.000	0.022	0.001	0.265
130	0.334	0.003	0.334	0.211	0.055	1.000	0.997	0.055	0.093	0.997
170	0.733	0.602	0.167	0.558	0.118	0.024	0.104	0.849	1.000	0.882

Table S5. Comparison of the swelling ratios between microspheres after the immersion into pH 6.5 PBS and pH 7.4 PBS from 0.5 to 24 h within each group with a specific alginate:$CaCl_2$ ratio.

	p Value (pH 6.5 vs. pH 7.4, *t*-Test)		
**Time (h)**	**4:1**	**2:1**	**1:1**
0.5	0.000	0.000	0.000
1.0	0.000	0.001	0.000
1.5	0.000	0.000	0.000
2.0	0.000	0.000	0.000
12	0.000	0.000	0.000
24	0.000	0.000	0.000

Table S6. Comparison of the swelling rates of microspheres between groups (with different alginate:$NaHCO_3$ ratios) after the immersion into pH 6.5 PBS and pH 7.4 PBS from 0.5 to 24 h.

	p Value (ANOVA/Scheffe Test)					
	**4:1 vs. 2:1**		**4:1 vs. 1:1**		**2:1 vs. 1:1**	
**Time (h)**	**pH 6.5**	**pH 7.4**	**pH 6.5**	**pH 7.4**	**pH 6.5**	**pH 7.4**
0.5	0.018	0.655	0.008	0.000	0.767	0.000
1.0	0.007	0.729	0.000	0.000	0.032	0.001
1.5	0.000	0.232	0.000	0.000	0.019	0.000
2.0	0.023	0.075	0.001	0.001	0.051	0.007
12	0.027	0.419	0.000	0.001	0.011	0.003
24	0.006	0.662	0.000	0.047	0.002	0.147

Table S7. Comparison of the average pH values between microsphere in PBS and PBS containing 10% FBS in different groups with different alginate:$NaHCO_3$ ratios after the immersion from day 1 to day 22.

	p Value (PBS vs. PBS + FBS, *t*-Test)		
**Day**	**4:1**	**2:1**	**1:1**
1	0.008	0.002	0.022
3	0.171	0.010	0.076
5	0.011	0.011	0.016
7	0.014	0.630	0.294
10	0.292	0.008	0.002
14	0.019	0.010	0.001
18	0.010	0.785	0.014
22	0.000	0.001	0.000

Table S8. Comparison of the average pH values among microspheres with different alginate:$NaHCO_3$ ratios in PBS and PBS containing 10% FBS after the immersion from day 1 to day 22 .

	***p* Value (ANOVA/Scheffe Test)**					
	**4:1 vs. 2:1**		**4:1 vs. 1:1**		**2:1 vs. 1:1**	
**Day**	**PBS**	**PBS + FBS**	**PBS**	**PBS + FBS**	**PBS**	**PBS + FBS**
1	0.006	0.026	0.000	0.000	0.000	0.000
3	0.900	0.112	0.000	0.000	0.000	0.002
5	0.318	0.014	0.006	0.000	0.001	0.000
7	0.061	0.010	0.000	0.000	0.000	0.000
10	0.003	0.000	0.000	0.000	0.009	0.000
14	0.003	0.001	0.000	0.000	0.000	0.004
18	0.006	0.000	0.000	0.000	0.003	0.000
22	0.001	0.000	0.000	0.000	0.002	0.000

Table S9. Comparison of the accumulated Dox release rates between microspheres in PBS and PBS containing 10% FBS in different groups (ratio = alginate:$NaHCO_3$) from day 1 to day 22 after immersion.

	***p* Value (PBS vs. PBS + FBS, *t*-Test)**		
**Day**	**4:1**	**2:1**	**1:1**
1	0.000	0.000	0.000
3	0.000	0.000	0.000
5	0.000	0.000	0.000
7	0.000	0.000	0.000
10	0.000	0.000	0.000
14	0.000	0.000	0.000
18	0.000	0.000	0.001
22	0.000	0.000	0.000

Table S10. Comparison of the accumulated Dox release rates among microspheres in PBS and PBS containing 10% FBS (ratio = alginate:$NaHCO_3$) from day 1 to day 22.

	***p* Value (ANOVA/Scheffe Test)**					
	**4:1 vs. 2:1**		**4:1 vs. 1:1**		**2:1 vs. 1:1**	
**Day**	**PBS**	**PBS + FBS**	**PBS**	**PBS + FBS**	**PBS**	**PBS + FBS**
1	0.000	0.000	0.000	0.000	0.015	0.001
3	0.000	0.000	0.000	0.000	0.001	0.001
5	0.000	0.000	0.000	0.000	0.007	0.000
7	0.000	0.005	0.000	0.000	0.001	0.003
10	0.008	0.032	0.000	0.000	0.002	0.003
14	0.002	0.001	0.000	0.000	0.000	0.004
18	0.021	0.111	0.000	0.011	0.001	0.197
22	0.040	0.001	0.000	0.000	0.001	0.005

Table S11. Model summary of multiple linear regression analysis used to evaluate the effects of Dox release amount (µg) and the environmental pH value on cell viabilities.

Summary of Cell Viability Model				
	**R**	**R Square**	**Adjusted R Square**	**Standard Error of the Estimate**
**Huh-7**				
Day 4	0.850	0.723	0.686	4.620
Day 8	0.952	0.906	0.893	4.559
Day 12	0.979	0.958	0.953	1.724
**Huh-3B**				
Day 4	0.891	0.794	0.766	3.411
Day 8	0.987	0.975	0.972	1.606
Day 12	0.940	0.883	0.868	3.818

Table S12. Analysis of variance (ANOVA) showed the regression, residual, F and *p* values after treatments with Dox/$NaHCO_3$ calcium alginate microspheres for 4, 8 and 12 days.

ANOVA of Cell Viability Model						
**Cells**		**SS**	**df**	**MS**	***F***	***p* Value**
**Huh-7**						
Day 4	Regression	834.910	2	417.455	19.556	0.000
	Residual	320.201	15	21.347		
	Total	1155.111	17			
Day 8	Regression	2989.191	2	1494.596	71.912	0.000
	Residual	311.753	15	20.784		
	Total	3300.944	17			
Day 12	Regression	1023.023	2	511.512	172.081	0.000
	Residual	44.588	15	2.973		
	Total	1067.611	17			
**Hep-3B**						
Day 4	Regression	672.555	2	336.278	28.897	0.000
	Residual	174.556	15	11.637		
	Total	847.111	17			
Day 8	Regression	1514.240	2	757.120	293.422	0.000
	Residual	38.705	15	2.580		
	Total	1552.944	17			
Day 12	Regression	1657.338	2	828.669	56.846	0.000
	Residual	218.662	15	14.577		
	Total	1876.000	17			

SS: Sum of squares, **df:** Degree of freedom, **MS:** Mean square, **F:** F test.

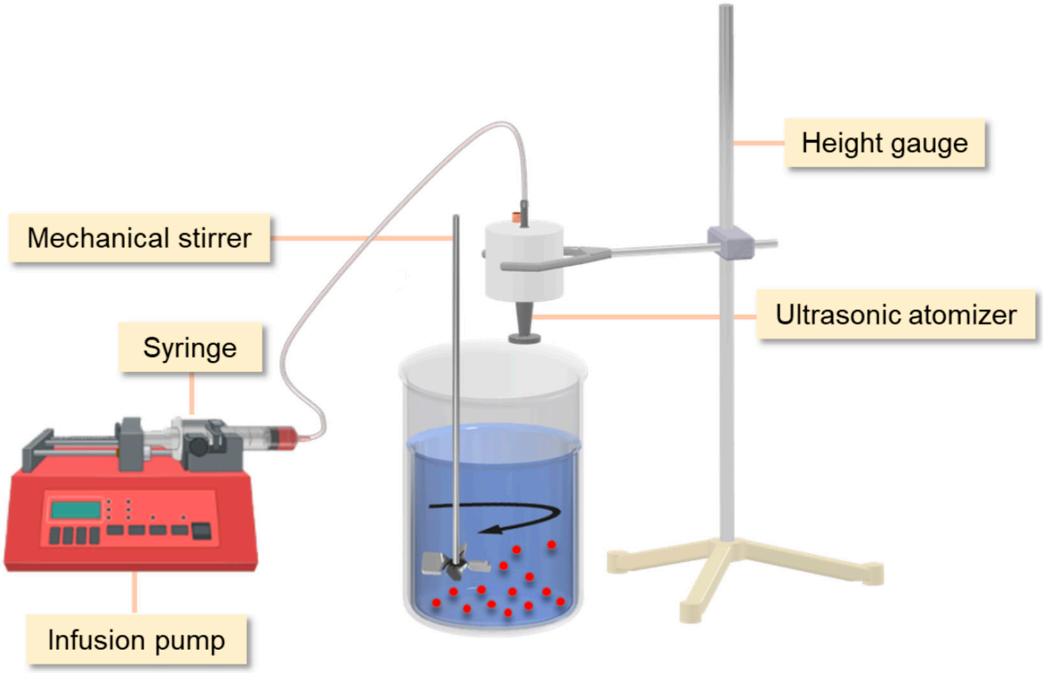

Figure S1. The experimental setups where the mixture (sodium alginate, $NaHCO_3$ and/or Dox) was located in a syringe and the syringe was squeezed by an infusion pump.

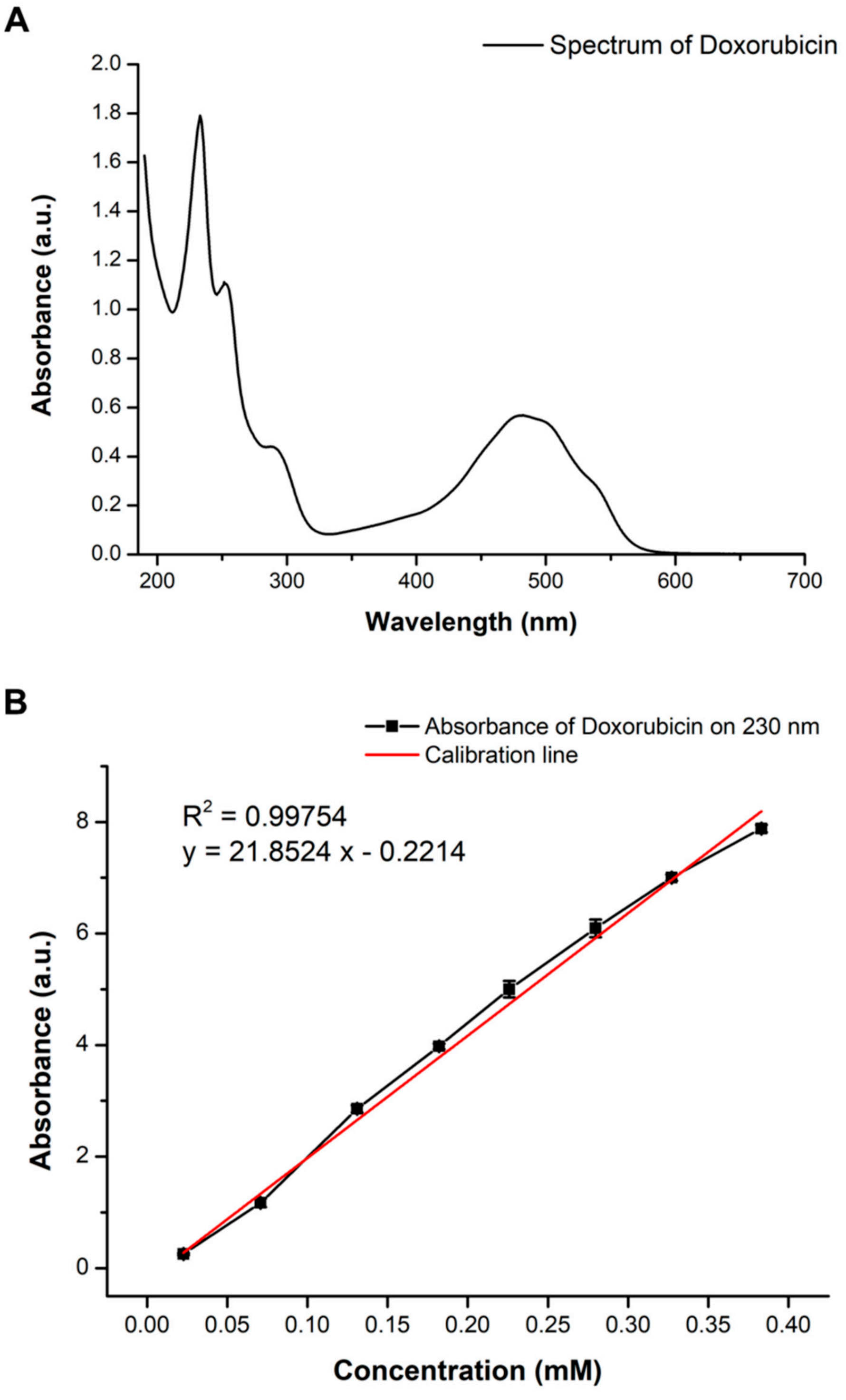

Figure S2. A calibration curve was generated to detect Dox concentration. **(A)** Dox exhibited a notable peak at ~230 nm by using a broadband spectrophotometer. **(B)** A linear calibration curve was generated by series of dilutions with sterile MiniQ ultrapure water, 0.375, 0.325, 0.275, 0.225, 0.175, 0.125, 0.075 and 0.025 mM, respectively, and measured at optical absorbance 230 nm (OD_{230}).

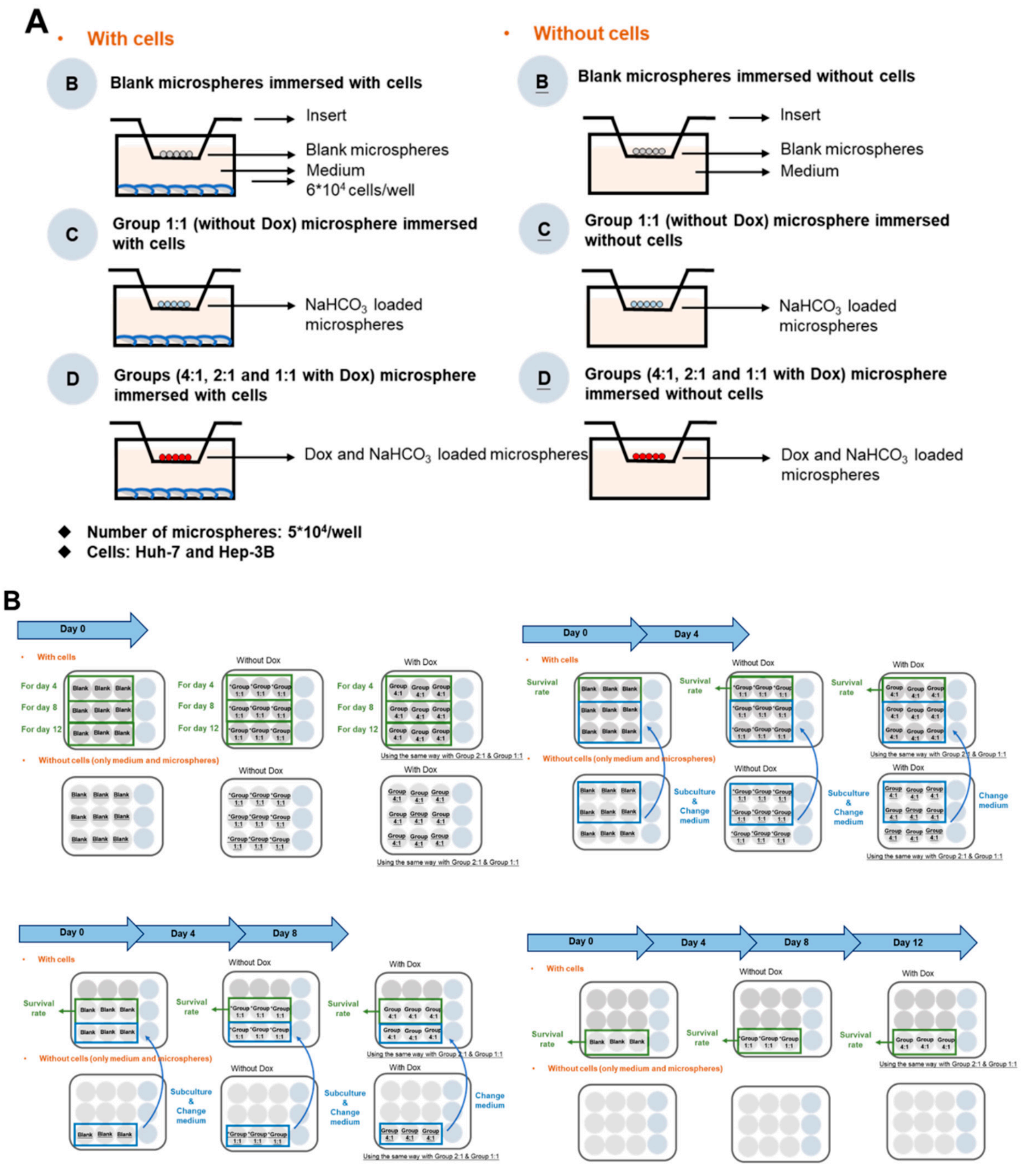

Figure S3. Media with the cumulative Dox concentrations were replaced every 4 days to reduce the experimental errors due to the depletion of nutrients in the media. (**A**) Cells (6 × 10⁴) were seeded in a 12-well plate overnight and treated with 5 × 10⁴ microspheres (without or with the Dox) in the cell culture insert to specifically separate cells from the microspheres, yet containing sufficient media to cover the microspheres for drug release. (**B**) After cell viabilities were determined on days 4 and 8, the remaining unmeasured well plates were replaced with media without cells.
